# Inhibition of full length Hepatitis C Virus particles of 1a genotype through small interference RNA

**DOI:** 10.1186/1743-422X-8-203

**Published:** 2011-05-02

**Authors:** Muhammad Ansar, Usman Ali Ashfaq, Imran shahid, Muhammad Tahir Sarwar, Tariq Javed, Sidra Rehman, Sajida Hassan, Sheikh Riazuddin

**Affiliations:** 1 Division of Molecular Medicine, National Centre of Excellence in Molecular Biology, University of the Punjab, Lahore, Pakistan; 2 Department of Human Genetics, Radboud University Nijmegen Medical Centre, Nijmegen, The Netherlands; 3 Applied and Functional Genomics Laboratory, National Center of Excellence in Molecular Biology, University of Punjab, Lahore, Pakistan; 4 Allama Iqbal Medical College, Allama Shabir Ahmad Usmani Road, Lahore, Pakistan

## Abstract

**Background:**

Hepatitis C virus (HCV), a member of the *Flaviviridae *family of viruses, is a major cause of chronic hepatitis, liver cirrhosis and hepatocellular carcinoma. Currently, the only treatment available consists of a combination of Pegylated interferon alpha (INF-α) and ribavirin, but only half of the patients treated show a sufficient antiviral response. Thus there is a great need for the development of new treatments for HCV infections. RNA interference (RNAi) represents a new promising approach to develop effective antiviral drugs and has been extremely effective against HCV infection.

**Results:**

This study was design to assess or explore the silencing effect of small interference RNAs (siRNAs) against full length HCV particles of genotype 1a. In the present study six 21-bp siRNAs were designed against different regions of HCV structural genes (Core, E1 and E2). Selected siRNAs were labeled as Csi 301, Csi 29, E1si 52, E1si 192, E2si 86 and E2si 493. Our results demonstrated that siRNAs directed against HCV core gene showed 70% reduction in viral titer in HCV infected liver cells. Moreover, siRNAs against E1 and E2 envelop genes showed a dramatic reduction in HCV viral RNA, E2si 86 exhibited 93% inhibition, while E1si 192, E2si 493 and E1si 52 showed 87%, 80%, and 66% inhibition respectively. No significant inhibition was detected in cells transfected with the negative control siRNA.

**Conclusion:**

Our results suggested that siRNAs targeted against HCV structural genes efficiently silence full length HCV particles and provide an effective therapeutic option against HCV infection.

## Background

HCV was identified in 1989 as the leading pathogen for non-A, non-B viral hepatitis [1]. HCV is an enveloped positive-single stranded RNA virus 9.6 kb in length consisting of structural (Core, E1, E2 and possibly p7) proteins and nonstructural (NS2, NS3, NS4A, NS4B, NS5A and NS5B) proteins [2,3]. HCV Core is known as the inducer of steatosis, oxidative stress and liver cancer [4]. E1 and E2 are involved in virus attachment with the cells and are considered to be the first viral proteins come in contact with the cells [5]. An estimated more than 170 million individuals worldwide have been chronically infected, while 3-4 million new infections believed to occur each year [6]. Many infected individuals develop liver damage with an increased risk of progression to fibrosis, cirrhosis, and liver cancer [7].

Currently the standard therapy for HCV is pegylated interferon (PEG-INF) with nucleoside analog ribavirin (RBV). This therapy achieves 50% sustained virological response (SVR) for genotype 1, which is the most prevalent form of the virus in the United States, Western Europe and Japan. SVR is 80% for genotype 2 & 3, which is the most prevalent genotype in Pakistan [8-10]. A sustained viral response occurs when there is no trace of HCV RNA present in the patient's blood immediately after treatment and also six months post-treatment. As pegylated interferon is expensive, standard interferon is still the main therapy for HCV treatment in under developed countries. Studies showed that current therapies are costly and cause a variety of side effects, including irritability, headache, flu-like symptoms, anemia, depression and gastrointestinal symptoms [10]. Low response rates and the significant side effect burden of current HCV therapies necessitate the identification of more effective anti-HCV agents, especially for treatment of patients infected with genotype 1a.

RNA interference (RNAi), a post-transcriptional regulation mechanism, is initiated by small interfering RNAs (siRNAs) of 21-23 nucleotides, which are incorporated into a multi-protein complex commonly known as the RNA-induced silencing complex (RISC), leading to sequence-specific degradation of target mRNA recognized by the antisense strand of the siRNA [11-16]. RNAi was first discovered in the nematode worm *Caenorrhabditis elegans *[11], but it is present in many other organisms such as *Drosophila*, certain parasitic protozoa, and vertebrates [17,18]. Small interference RNA (siRNA) is a valuable tool to inhibit the expression of a target gene in a sequence-specific manner, and may be used for functional genomics, target validation and therapeutic purposes. The difference between antisense approaches and conventional drugs is that the conventional drugs bind to proteins and thereby modulate their function whereas antisense agents act at the mRNA level, preventing its translation into protein [19,20]. siRNAs can be used as a potential therapeutic agents against HCV because HCV replication takes place in the cytoplasm of liver cells without integration into the host genome. Moreover, its genome functions both as an mRNA and as a replication template. Several reports demonstrated potent RNAi activity against HCV in sub-genomic replicon and infection [21-23]. Synthetic or vector based siRNAs targeted against 5' untranslated region (UTR), HCV core, NS3, NS4B and NS5B were found to be effective in reducing viral replication and infection [22-26].

The present study was undertaken to study the effect of siRNAs directed against the structural genes of the HCV genotype 1a in HCV infected liver cells. It demonstrates that the RNAi-mediated silencing of the HCV full length viral particle may be one of the important therapeutic opportunities against HCV 1a genotype.

## Materials and methods

### Serum Sample Collection

HCV-1a patient's serum samples used in this investigation were obtained from the CAMB (Center for Applied Molecular Biology) diagnostic laboratory, Lahore, Pakistan. Serum samples were stored at -80°C prior to viral inoculation experiments. Quantification and genotype was assessed by CAMB diagnostic laboratory, Lahore, Pakistan. Patient's written consent and approval for this study was obtained from institutional ethics committee.

### siRNAs designing

Small interfering RNA oligonucleotides against HCV core, envelop protein (E1 and E2) and P7 genes were designed to the most conserved target region of these genes using the Ambion's siRNA design tool http://www.ambion.com/techlib/misc/siRNA_finder.html. The designed siRNAs against HCV core, envelop protein (E1 and E2) and P7 genes were synthesized using Silencer siRNA construction kit according to the manufacturer's instruction (Ambion, USA).

### Cell line

The Huh-7 cell line was compassionately offered by Dr. Zafar Nawaz (Biochemistry and Molecular Biology Department, University of Miami, USA). Huh-7 cells were cultured in Dulbecco's modified Eagle medium (DMEM) supplemented with 10% fetal bovine serum & 100 IU/ml penicillin & 100 μg/ml streptomycin, at 37°C in an atmosphere of 5% CO_2_.

### MTT assay for toxicity

To investigate cellular toxicity, 2 × 10^4 ^cells/well was plated into 96-well plates. After 24 h, different concentrations of siRNAs were added and the plate was sealed and kept at 37°C in an atmosphere of 5% CO^2 ^for 24 h. After the siRNAs treatment were over, removed the media and siRNAs. About 100 μl fresh media and 20 μl of MTT solution (5 mg/ml in PBS) were added to all wells in Columns 1-11. Wrapped the plate in aluminum foil and incubated for 3-4 h at 37°C. Media was carefully removed and added 100 μl of DMSO to dissolve the formazan crystals in Columns 1-11. MTT formazan product was determined by measuring absorbance with an enzyme-linked immunosorbent assay (ELISA) plate reader at a test wavelength of 570 nm and a reference wavelength of 620 nm.

Cell viability was obtained using the following equation:

### Silencing effect of siRNAs in serum infected liver cells

Huh-7 cell line was used to establish the in-vitro replication of HCV. A similar protocol was used for viral inoculation as established by Zekari et al. 2009 [27] and El-Awardy et al. 2006 [28]. High viral titer >1 × 10^8 ^IU/ml from HCV-1a patient's was used as principle inoculum in these experiments. Huh-7 cells were maintained in 6-well culture plates to semi-confluence, washed twice with serum-free medium, then inoculated with 500 μl (5 × 10^7^IU/well) and 500 μl serum free media. Cells were maintained overnight at 37°C in 5% CO_2_. Next day, adherent cells were washed three times with 1 × PBS, complete medium was added and incubation was continued for 48 h. Cells were harvested and assessed for viral RNA quantification by Real Time PCR. To analyze the effect of siRNAs on HCV infection, serum infected Huh-7 cells were again seeded after three days of infection in 24-well plates in the presence and absence of siRNAs and grown to 80% confluence. After 48 h, cells and total RNA was isolated by using Gentra RNA isolation kit (Gentra System Pennsylvania, USA) according to the manufacturer's instructions. Briefely, cells were lysed with cell lysis solution containing 5 μl internal control (Sacace Biotechnologies Caserta, Italy). RNA pallet was solubilized in 1% DEPC (Diethyl pyrocarbonate treated water). HCV RNA quantifications were determined by Real Time PCR Smart Cycler II system (Cepheid Sunnyvale, USA) using the Sacace HCV quantitative analysis kit (Sacace Biotechnologies Caserta, Italy) according to the manufacturer's instructions.

### Formula for the calculation of HCV RNA concentration

Following formula was used to calculate the concentration HCV RNA of each sample.

IC = internal control, which is specific for each lot.

### Statistical Analysis

All statistical analysis was done using SPSS software (version 11.0, SPSS Inc). Data is presented as mean ± SE. Numerical data was analyzed using student's t-test and ANOVA. P value < 0.05 was considered statistically significant.

## Results

### Toxicological analysis of siRNAs

**Figure 1:**
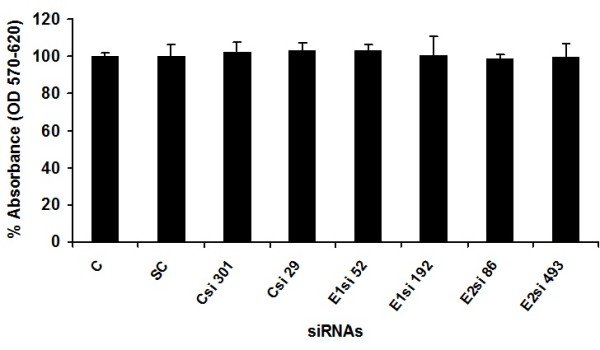
**Toxicity of siRNAs through MTT cell proliferation assay**. Huh-7 cells were plated at the density of 2 × 10^4 ^in 96 well plates. After 24 h cells were treated with different concentrations of siRNAs and control consisted of solvent in which siRNAs dissolved. After 24 h incubation period add MTT solution to all wells and incubated for 3-4 h at 37°C. Viable cells convert MTT to purple formazan crystal. Added DMSO to dissolve the formazan crystals and read absorbance at 570 nm and 620 nm.

Before starting the antiviral screening against HCV, toxicological effect of siRNAs were determined through MTT cell proliferation assay. The MTT substance is reduced by mitochondrial succinic dehydrogenases in living cells to purple formazan crystals that are not soluble in aqueous water. The absorption of dissolved formazan in the visible region correlates with the number of alive cells [29]. Figure 1 shows cytotoxicity analysis of siRNAs and demonstrates that Huh7 cells viability is unaffected at a concentration of 20 μM (Figure 1).

### Silencing effect of siRNAs on liver infected cells

**Figure 2:**
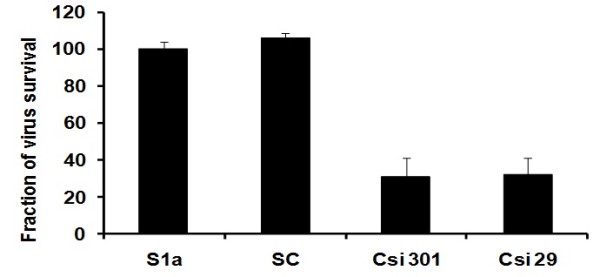
**Silencing effect of core specific siRNAs against full length viral particles of HCV 1a genotype**. Huh-7 cells were infected with high titer sera sample from HCV-1a patients (S1a) to establish in vitro cell culture model of HCV-1a, cells were maintained overnight at 37°C in 5% CO2 for three days. Cells were harvested after siRNA treatment 48 hrs post transfection and intracellular HCV RNA levels were quantified by Real Time PCR. Data is expressed as percentage of HCV survival in cells. S1a means control consisted of HCV serum of 1a genotype) and SC means scrambled siRNA. Error bars indicate, mean S.D p < 0.05 verses S1a.

**Figure 3:**
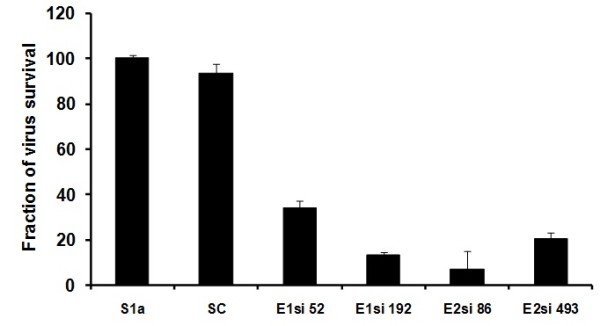
**Silencing effect of HCV E1 and E2 specific siRNAs against full length viral particles of HCV 1a genotype**. Huh-7 cells were infected with high titer sera sample from HCV-1a patients (S1a) to establish in vitro cell culture model of HCV-1a, cells were maintained overnight at 37°C in 5% CO2 for three days. Cells were harvested after siRNA treatment 48 hrs post transfection and intracellular HCV RNA levels were quantified by Real Time PCR. Data is expressed as percentage of HCV survival in cells. S1a means control consisted of HCV serum of 1a genotype) and SC means scrambled siRNA. Error bars indicate, mean S.D p < 0.05 verses S1a.

The ability of siRNAs to inhibit HCV replication and infection was evaluated by designing and constructing siRNAs against different sites of HCV structural genes having genotype1a (Core, E1 and E2). siRNAs targeting sites were selected in regions conserved among different samples. Selected siRNAs were labeled as Csi 301, Csi 29, E1si 52, E1si 192, E2si 86 and E2si 493. Negative control siRNA (scrambled siRNA) with the same nucleotide composition as the experimental siRNA which lacks significant sequence homology to the HCV and human genome was designed (Table 1). The results indicate that siRNAs targeting HCV structural genes have the ability to inhibit the whole virus of 1a genotype in serum infected liver cells. The effect of gene-specific siRNAs against the whole virus was evaluated in serum-infected Huh-7 cells by measuring reduction in the HCV RNA titer. Real-time PCR results showed that core siRNAs (Csi 301 and Csi 29) reduced HCV-1a RNA level up to 70% (Figure 2). siRNAs against E1 and E2 envelop genes showed a dramatic reduction in HCV viral RNA, E2si 86 showed a maximum inhibition of about 93%, while E1si 192, E2si 493 and E1si 52 showed 87%, 80%, and 66% inhibition respectively (Figure 3). Together, these data suggest a negative impact of chemically synthesized Core and envelop genes siRNAs on HCV replication and infection that could be used for the down regulation of the whole viral particle of HCV la genotype. This result was in accordance with Zekri et al. 2009 [27] who also showed best inhibitory effect of siRNAs against 5'UTR on 3rd day of post-transfection.

**Table 1:** Sequence of siRNA oligonucleotides directed against structural genes of HCV 1a genotype

SN	siRNAs Name	Sequence 5'-3'
1	Csi 29-antisense	AAACCAAACGTAACACCAACCCCTGTCTC
2	Csi 29-sense	AAGGTTGGTGTTACGTTTGGTCCTGTCTC
3	Csi 301-antisense	AAGGTCATCGATACCCTTACGCCTGTCTC
4	Csi 301-sense	AACGTAAGGGTATCGATGACCCCTGTCTC
5	E1si 52-antisense	AACTCGAGTATTGTGTACGAGCCTGTCTC
6	E1si 52-sense	AACTCGTACACAATACTCGAGCCTGTCTC
7	E1si 192-antisense	AACGCAGCTTCGACGTCATATCCTGTCTC
8	E1si 192-sense	AAATATGACGTCGAAGCTGCGCCTGTCTC
9	E2si 86-antisense	AACTGATCAACACCAACGGCACCTGTCTC
10	E2si 86-sense	AATGCCGTTGGTGTTGATCAGCCTGTCTC
11	E2si 493-antisense	AATTGGTTCGGTTGTACCTGGCCTGTCTC
12	E2si 493-sense	AACCAGGTACAACCGAACCAACCTGTCTC

## Discussion

HCV is a leading cause of acute and chronic hepatitis which can eventually lead to permanent liver damage, hepatocellular carcinoma and death. Currently, there is no vaccine available for prevention of HCV infection due to high degree of strain variation. The only therapy available is a combination of interferon and ribavirin, which cure only 50% of patients having genotype 1a. RNA interference (RNAi) represents a new promising approach to develop effective antiviral drugs against HCV. In order to identify potent anti-HCV agents, many investigators design and test multiple oligonucleotides that target different sites and regions of the target mRNA and many of them show desired results [30]. The underlying mechanism of siRNA-mediated gene silencing is still unknown in mammalian cells; it could interfere with RNA stability and/or translation or, alternatively, transcription. In cultured mammalian cell lines, chemically synthesized siRNAs can be introduced into cells when formulated with lipophilic reagents [31]. Longer dsRNAs (50 bp) have a more broad effect in mammalian somatic cells, resulting in general arrest of protein synthesis through interferon response and also protein kinase activation. In contrast, shorter siRNAs of 21-23 nt have a more specific effect, inducing up to 90% suppression of specific mRNAs both *in vitro *and *in vivo *[12,22]. Due to its high suppression efficiency and sequence specificity up to a single nucleotide resolution [13] has encouraged the development of RNAi-based therapeutic models for possible use in viral infections i.e. HIV-1 [32], HBV [22], HCV [22], respiratory viruses [33] and cancer i.e. K-ras [34], PI 3-kinase [31]. The HCV genome is a positive-sense single-stranded RNA that functions as both a messenger RNA and replication template via a negative-strand intermediate, making it an attractive target for the study of RNA interference.

A potential problem that may arise in RNAi based approach is the error prone nature of HCV genome with generation of quasispecies during chronic HCV infection but this problem can be overcome by designing siRNAs against highly conserved region of HCV [35-37]. Several reports demonstrated that RNAi, efficiently inhibits viral replication and infection by targeting 5'UTR, Core, E1, E2, NS3, NS4b and NS5b sequences as alternative anti-HCV strategies [23,25,35,38-40]. Two different groups used siRNA against Core gene of HCV 1a and 1b genotype and observed 60% and 80% reduction in mRNA and protein expression respectively [40,41]. A study demonstrated that siRNAs targeted against E2, NS3 and NS5B regions effectively inhibit core gene expression [41] and Kim et al., 2006, has designed siRNAs against HCV 1b and 1a genome to explore the silencing of structural genes and showed significantly less expression in a dose-dependent manner. In the present study, six different siRNAs were designed, with GC content of 35-50%, targeting structural genes of HCV 1a genotype.

The plaque assay is the most important procedure in virology to measure virus infectious titers by visualizing the viral-induced cytopathic effect. However, plaque assay for HCV is not available because HCV is non-cytopathic and detection of HCV-infected cells commonly rely on visualization of the infected cells by immunostaining of HCV proteins [42], western blotting and RT PCR. Recently, different groups have studied HCV replication in serum-infected liver cell lines, which mimics the naturally occurring HCV infection in humans [28,43,44]. In this study, Huh-7 cells were infected with native viral particles from HCV-genotype-1a positive serum, using the same protocol as described by El-Awady et al., and Zekri et al. [27,28]. siRNAs against HCV-1a structural genes used in the present study were screened against HCV-serum-infected Huh-7 cells. An exciting finding of this study was a decrease in the HCV viral titer to a maximum of 93% after treatment with gene-specific siRNAs (E1si 88). siRNAs against HCV core gene (Csi 301, Csi 29) showed up to 70% inhibition in the viral titer of HCV 1a genotype (Figure 2). Similarly siRNAs directed against HCV envelop proteins (E1si 192, E2si 493 and E1si 52) showed 87%, 80%, and 66% inhibition in the viral titer of genotype 1a respectively (Figure 3). These results are in agreement with previous studies, which have suggested that siRNA is the most efficient nucleic-acid-based antiviral approach that can be utilized to degrade the HCV genome in infected cells.

In conclusion, current therapy for HCV infection is mainly the combination use of interferon and ribavirin, but only about half of the treated patients obtain a sustained antiviral response. Our data suggest that siRNAs targeting HCV structural genes can target viral RNA in infected cells, potentially providing an efficient therapeutic option against HCV infection.

## Abbreviations

**HCV**: Hepatitis C virus; **PEG-INF**: Pegylated interferon; **SVR**: Sustained Virological Response; **siRNAs**: Small interference RNAs; **Huh-7**: Human Hepatoma Cell line.

## Competing interests

The authors declare that they have no competing interests.

## Authors' contributions

MA, UAA and MI contributed equally in lab work and manuscript write up. MTS, TJ and SDR helped me in writing the manuscript. SH and SRD was the principal investigator and provides all facilities to complete this work. All the authors read and approved the final manuscript.

## Authors' information

Muhammad Ansar (PhD Scholar), Usman Ali Ashfaq (PhD Molecular Biology), Imran shahid (M Phil Molecular Biology), Muhammad Tahir Sarwar (PhD scholar), Tariq Javed (M. Phil pharmaceutical chemistry, Sidra Rehman (MSc Chemistry), Sajida Hassan (PhD Molecular Biology) and Sheikh Riazuddin (PhD molecular Biology and Dean Post graduate study at Allama Iqbal medical college, Lahore
